# National Mesothelioma Virtual Bank: A Platform for Collaborative Research and Mesothelioma Biobanking Resource to Support Translational Research

**DOI:** 10.1155/2013/765748

**Published:** 2013-09-18

**Authors:** Waqas Amin, Anil V. Parwani, Jonathan Melamed, Raja Flores, Arjun Pennathur, Federico Valdivieso, Nancy B. Whelan, Rodeny Landreneau, James Luketich, Michael Feldman, Harvey I. Pass, Michael J. Becich

**Affiliations:** ^1^Department of Biomedical Informatics, University of Pittsburgh Medical Center, Pittsburgh, PA, USA; ^2^Department of Pathology, University of Pittsburgh Medical Center, Pittsburgh, PA, USA; ^3^Department of Pathology, New York University Medical Center, New York, NY, USA; ^4^Department of Cardiothoracic Surgery, Mount Sinai School of Medicine, New York, NY, USA; ^5^Department of Cardiothoracic Surgery, University of Pittsburgh Medical Center, Pittsburgh, PA, USA; ^6^Department of Pathology, University of Pennsylvania, Philadelphia, PA, USA; ^7^Department of Cardiothoracic Surgery, New York University Medical Center, New York, NY, USA

## Abstract

The National Mesothelioma Virtual Bank (NMVB), developed six years ago, gathers clinically annotated human mesothelioma specimens for basic and clinical science research. During this period, this resource has greatly increased its collection of specimens by expanding the number of contributing academic health centers including New York University, University of Pennsylvania, University of Pittsburgh Medical Center, and Mount Sinai School of Medicine. Marketing efforts at both national and international annual conferences increase awareness and availability of the mesothelioma specimens at no cost to approved investigators, who query the web-based NMVB database for cumulative and appropriate patient clinicopathological information on the specimens. The data disclosure and specimen distribution protocols are tightly regulated to maintain compliance with participating institutions' IRB and regulatory committee reviews. The NMVB currently has over 1120 annotated cases available for researchers, including paraffin embedded tissues, fresh frozen tissue, tissue microarrays (TMA), blood samples, and genomic DNA. In addition, the resource offers expertise and assistance for collaborative research. Furthermore, in the last six years, the resource has provided hundreds of specimens to the research community. The investigators can request specimens and/or data by submitting a Letter of Intent (LOI) that is evaluated by NMVB research evaluation panel (REP).

## 1. Introduction

The National Mesothelioma Virtual Bank (NMVB) was developed in 2006 to stimulate the good of mesothelioma patients, collaborative efforts are made to collect and distribute well-annotated high-quality mesothelioma specimens to facilitate the research utilizing these specimens and encouraging basic, translational, and developmental studies [1]. The NMVB is funded by the Center of Disease Control and Prevention (CDC) in association with the National Institute of Occupational Health and Safety (NIOSH) [2, 3]. There are four academic health centers which have New York University School of Medicine (NYU), University of Pennsylvania (U. Penn), Mount Sinai School of Medicine (MSSM), and University of Pittsburgh Medical Center (UPMC) [2, 4–6]. With the collective efforts, NMVB is devoted to provide specimens from more than 1100 mesothelioma prospective and retrospective consented cases. NMVB provides fresh frozen, paraffin embedded blood products and three distinct tissue microarrays (TMA) to facilitate the areas in mesothelioma research that includes biomarkers study, immunology, genetics, and therapeutics.

The main objective of the NMVB is to provide a biorepository that can provide annotated specimens that are challenging to facilitate the initiation and performance of the research. The NMVB has addressed and overcome the challenges of most of the tissue banks are affected by providing sufficient number of mesothelioma cases, standardizing the data and specimen collection method, facilitating the sharing of information through NMVB database, and addressing the issues of constraint by confidentiality and de-identification.

The collaborative efforts of four academic institutions provide a unique opportunity for sharing clinical, pathological, and follow-up data on mesothelioma patients, while sharing the logistics of informatics and collaborative research programs across the cancer research institutions. The NMVB resource provides a virtual repository of mesothelioma specimens located at the collaborative sites. Each collaborative site keeps the autonomy of specimens, and information is shared through a well-standardized informatics platform, in order to make them available for mesothelioma research community. 

## 2. Methods

### 2.1. NMVB Collaborative Institutions

The NMVB resource is developed to provide the platform for sharing the well-annotated and highly characterized mesothelioma specimens for the mesothelioma research community for establishing collaborative research programs at the national level. We have envisioned that our experience in the development and deployment of tissue banking infrastructure will serve as a model for establishing tumor banks and collaborative research that will ultimately foster the translational research and provide highly characterized and well-annotated specimens to the research community. 

The NMVB resource was developed by the collaboration of the University of Pittsburgh Medical Center, New York School of Medicine, and the University of Pennsylvania [2, 4, 5]. The resource has successfully served the mesothelioma research community and fulfilled the needs of researchers by providing hundreds of mesothelioma specimens. After successful completion of five years of funding, the resource was refunded in 2011 with the spirit of expanding the collaboration and serving the research community by providing mesothelioma specimens, in order to facilitate the basic and clinical science research that will ultimately provide the benefit to the patients. In the second phase of funding, Mount Sinai School of Medicine joined the NMVB resource in 2011 [6]. With the expansion, the resource gained access to mesothelioma cases and research facilities from four medical care settings that are performing a vital role in the research and management of mesothelioma patients.

### 2.2. NMVB Resource Organization

**Figure 1:**
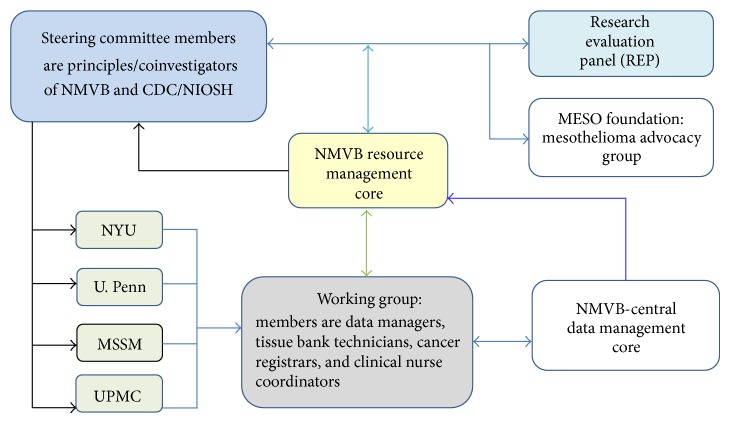
Presenting the organization of resource and collaborative efforts of expert domains to run the NMVB successfully. CDC (Center of Disease Control and Prevention), NIOSH (National Institute of Occupational Health and Safety), NYU (New York University), U. Penn (University of Pennsylvania), MSSM (Mount Sinai School of Medicine), and UPMC (University of Pittsburgh Medical Center).

National Mesothelioma Virtual Bank resource organization consists of the following three major elements (Figure 1). 

#### 2.2.1. Research Evaluation Panel (REP)

The Research Evaluation Panel (REP) is an independent group of experts that is created by the NMVB Steering Committee in consultation with the CDC program officer. The selection of REP members is based on their qualification, publications, and research experience in the mesothelioma research. The REP member could be a pathologist, biostatistician, physician, or an investigator that has a deep understanding of mesothelioma and has an up to date knowledge of recent advancement in the diagnosis and management of mesothelioma. The REP members work independently but collaboratively with the Coordinating Committee and without conflict of interests with the resource. They are responsible for reviewing requests from investigators for specimens and clinical data and evaluating the scientific merit of the request. The REP members then provide advice about the scientific quality and priority of the request to the resource NMVB Steering Committee. There are also nonvoting members who are members of NMVB Steering Committee who also provide their input on the specimen and data requests. 

REP members are selected for four years and the inclusion of new members/replacement is established with the consultation of the existing REP members, steering committee members, and CDC program officer. The REP chooses one of its members to be chairperson for a term of one to two years. The REP must be consistent with operating policies developed by the Steering Committee. The REP members will attend at least one Steering Committee meeting each year to help coordinate REP activities with those of the Steering Committee.

#### 2.2.2. Coordinating Committee

 The Coordinating Committee is comprised of the Principal Investigators from collaborative sites UPMC, NYU, U. Penn, and MSSM and program managers from Mesothelioma Allied Research Foundation (Meso-foundation), CDC, and the National Institute of Occupational Safety and Health (NIOSH) [2, 3]. 

The Coordinating Committee act as the governing body of the resource for developing operating policies that are implemented at each collaborative by their principle investigator. The Committee reviews the operating procedures of the participating sites to insure that they are compatible with the overall goals and policies of the resource and comply with CDC and NIOSH defined specific quality control and tissue processing procedures [2, 3]. The committee also overlooks the specimen requests that are approved by the REP and assure the fulfillment of requests as per resource protocols. The committee meets in person at least once a year with additional meetings as necessary. The committee conducts monthly conference calls. The meetings are aimed at coordinating the activities of the participating sites, establishing new policies and priorities, and reviewing progress. 

#### 2.2.3. NMVB Working Group

NMVB working group comprises of personnel from all collaborative sites that includes data managers, tissue bank technician, database administrator, cancer registrar, and research coordinator who handles day-to-day activities of the project. The working group meeting is conducted every month and project coordinator at the coordinator site reports, the progress to NMVB steering committee. 

The data managers are responsible for entering de-identified data of consented mesothelioma cases of their local site into the data entry interface of NMVB database. They are responsible for keeping and maintaining patient health information (PHI) according to Health Insurance Portability and Accountability Act (HIPAA) and local Institutional Review Board rules and regulations. The data manager of each collaborative site is the key personal that communicates regarding specimen collection and annotation with tissue bank technicians and cancer registry managers from their own institution. Data Managers will eventually be responsible for quality assurance standards within their center as they relate the data entered into the NMVB database.

### 2.3. Retrospective Collection

Retrospective collection of NMVB arise clinically collected specimens (fresh frozen and paraffin blocks) containing specimens from surgical resection. Pneumonectomy, pleural, and peritoneal resection, core needle biopsies, lymph nodes, and other biopsies or tissue specimens of metastatic mesothelioma sites are also collected. The local Institutional Review Board (IRB) waives the retrospective collection of specimen; NMVB sites may bank existing specimens without patient consent. However, if a patient is alive and the patient needs to be contacted in order to obtain additional clinical information, each site will do so according to a written plan approved by their Institutional Review Board. 

The honest broker searches mesothelioma cases in clinical information systems that are seen during the preceding year but are not consented due to various reasons. The patient denies consent for the study, either the consenting staff does not approach the patient or the collection site does not have research staff available at the time the patient visited for surgical or follow-up treatments. The Honest broker compares the list of non-consented cases in NMVB honest broker log sheet to eliminate the possibility of prior enrollment into NMVB. The list of nonconsented patients is sent to NMVB principle investigator, clinical team (surgeons, physicians, and nurses), and research nurse coordinator. The clinical team reviews the patient list for a vital status “alive” and approaches their patients who were contacted via mailed letter in person or by phone to solicit their interest in the study. If the letter is sent, the honest broker obtains an electronic signature from each respective surgeon and sends a letter to the patient with study materials including either a response card to the study coordinator and/or phone number. The study coordinator and honest broker keep track of the patient consenting to NMVB with clinical team. The honest broker follows up with the clinical team periodically in the hope of improving the volume of participants. The selected cases are then sent to the tumor bank at each site so, the paraffin or fresh frozen samples can be collected from the clinical pathology department. The tissue bank makes a request to the pathology department to provide specimens for research purposes that are surplus to the clinical diagnostic use. Upon the receipt of specimens, the honest broker enrolls the patient into NMVB database and collects data at the patient's demographics, clinical pathology, and follow-up levels. The designated pathologist reviews the section slides of the case and completes the specimen and block level information in NMVB database. 

### 2.4. Prospective Collection

The participants are approached on their clinical visit at each collaborative site for the purpose to enroll in NMVB study by the research nurse coordinators or physicians. The clinical research nurse coordinator asks the participants to review a copy of the informed consent form (National Mesothelioma Virtual Registry and Tissue Bank (NMVRTB) Consent Form) prior to seeing their physicians. The clinical research nurse coordinators or physicians review the informed consent form with participants and address any questions or concerns prior to obtaining written informed consent for their biospecimens contribution to the NMVB study. They also address any future questions or concerns of NMVB participants and provide the patient with a NMVB Health Assessment Questionnaire that can be completed later at their convenience.

The participants are also informed that the research studies in which their donated specimens will be used will be directed at mesothelioma research. However, it is unlikely that their donated specimens are used in research studies directed at other diseases or conditions. The specimens from consented patients are stored at the tumor banks of each participating institute and de-identified information is shared through a web-based interface of NMVB database. After the participants undergo surgical treatment, tumor banks at each site collect the specimen and store them in the bank. The data manager enrolls the participant in the NMVB database and completes the patients de-identified demographic, epidemiology, pathology, and follow-up information.

### 2.5. Data Standardization and Management

To collect standardized data that can be uniform, consistent, understandable, and shareable, the Common Data Elements (CDE) were developed to allow annotation of cases and to accomplish characterization of biospecimens collected from different collaborating sites. The development of CDEs for the resource has been described in detail in a related publication from our group. In summary, the CDE development was by joint consensus of participants under the leadership supervision of the NMVB Coordinating Committee. The CDEs include cancer registry data (demographic, epidemiology, and follow-up) at the patient level, pathology data at the specimen level including data elements to elaborate TNM staging and tumor grading, along with block level annotation and genotype data. The CDE subcommittee used the experiences gained from previous projects and major standards used to develop the CDEs include the NAACCR Data Standards for Cancer Registries, CAP Cancer Protocol and Checklist, the Association of Directors of Anatomic and Surgical Pathology (ADASP), and the American Joint Committee on Cancer (AJCC) Cancer Staging Manual [4, 7–10]. 

The collected data from collaborative sites is reviewed at the NMVB coordinating (database) site, which processes the data according to quality assurance (QA) and quality check (QC) protocols quarterly that have been established by the resource. The process of QA/QC is performed to locate any missing datasets and errors performed during data recording. The value of each data set is matched with the CDE dictionary to make certain that the standards are being followed during data recording. Only the standardized data is uploaded into the database at regular basis, and data with errors is sent back to the corresponding sites along with explanations of rejection. The correction of the data is the responsibility of each collaborative site. The resource selects 5% of the newly entered cases to be reevaluated by honest brokers, cancer registrars, and data managers. After completion of their review, the audit reviewers submit a report of their findings and recommendations to the resource [11]. The resource members discuss their findings in the subsequent general meeting of the NMVB Coordinating Committee and make additional plans to implement the proposed recommendations.

The NMVB resource has periodic quality assurance and quality control of pathology data by assessing interobserver concordance for the resource pathologists. These consist of two methods which include (1) a joint review of cases at meetings and (2) an independent review of cases circulated amongst the participating sites. For joint QA review during meetings, resource pathologists review up to 5 cases from each site, with an emphasis on the 5 “matrix slides” selected by each pathologist at each site to include areas of difficulty or likely diagnostic differences. Joint review of cases is performed on a multiheaded microscope that permits pathologists to discuss diagnostic differences and set thresholds. Independent review of cases is performed by individual pathologist at each site on cases sent from the other collaborative sites at regular intervals. The data managers at each site will randomly select the cases for QA review from those added to the resource within certain cut-off dates. The review will be documented by completing the “matrix” fields and select, “histology” fields of the NMVB database. Any areas that show a high level of discrepancy will be determined by data managers and communicated to resource pathologists. The Pathology Subcommittee will then discuss their findings in the subsequent general meeting of the Coordinating Committee and provide a report with recommendations as indicated by their findings.

#### 2.5.1. De-Identification Utilizing the Honest Broker System

The NMVB has designed its procedures to protect the confidentiality and privacy of human subjects and IRB approval has been obtained for all activities. NMVB uses a decentralized system for specimen and data collection and storage. Each case is assigned a de-identified NMVB number. The only link to patient identity is held in reserve locally within the institution and there are no links directly connecting specimens or data to patients. The NMVB database is available to the research community on a public website but it is designed to produce only de-identified datasets upon query. This is possible because only de-identified data sets exist which are compliant with the “safe harbor” approach to HIPAA (Health Insurance Portability and Accountability Act) [12]. The “safe-harbor” approach entails the removal of all 18 identifiers enumerated at section 164.514(b) of the regulations [13]. The honest broker acts as a barrier between fully identified confidential clinical patient information and the completely de-identified data made available to the research community. An honest broker is an individual, organization, or system acting for or on behalf of the covered entity to collect and provide health information to the investigators in such a manner whereby it would not be reasonably possible for the investigators or others to identify the corresponding patients-subjects directly or indirectly. The honest broker cannot be one of the investigators or researcher. A researcher may use the services of an honest broker service to obtain the Protected Health Information in a de-identified manner. De-identification means that researchers or others cannot identify the patients-subjects directly or indirectly through identifiers link to the patient-subject. This honest broker service will de-identify medical records information by automated or manual methods. All honest broker services are approved in advance by both the IRB of record and collaborative sites of NMVB. The honest brokers are individuals who have clinical responsibilities as tissue bankers, data managers to manage the pathology data, or as cancer registry specialists in cancer registry. Based on their clinical job duties, their educational backgrounds and experiences vary. Depending on the nature of the projects, these bankers can work autonomously or collaboratively to meet biospecimens and/or data needs [14].

### 2.6. NMVB Database Architecture

The NMVB database is developed by utilizing a UML (spell it out) class diagram. Unified Modeling Language (UML) is a nonproprietary language for constructing, visualizing, and documenting the artifacts of software engineering [15]. The information model for CDEs is developed to establish a structurally aligned generalized relationship. The informatics model of database facilitates is achieving semantic and syntactic interoperability by describing the common data elements in the form of metadata or data descriptors and by using a controlled vocabulary in order to make the data understandable and sharable for end-users. The system architecture is designed to provide query speed and high security as well as expansion capabilities for incorporating new data elements or integrating existing systems at participating institutions.

The NMVB web-based query tool is based on the caTISSUE Clinical Annotation Engine (CAE) version 3.0 application [16]. The database provides three levels of access to end users, first, NMVB statistical data for public view, second, approved investigator database query that allows seeing individual patient de-identified clinical data, and third, data manager access to query and edit the stored data. Patient privacy is of utmost importance at all levels of user's access. The NMVB database allows researchers to search clinically annotated mesothelioma biospecimens via a web interface. The database is made available through a publicly available website. The database facilitates standardized clinical annotation structure and incorporates a variety of data sets from different data sources. Two methods of annotation have been adopted manually using web-based manual data entry tool and data imported electronically through excel files [1].

## 3. Results

**Figure 2:**
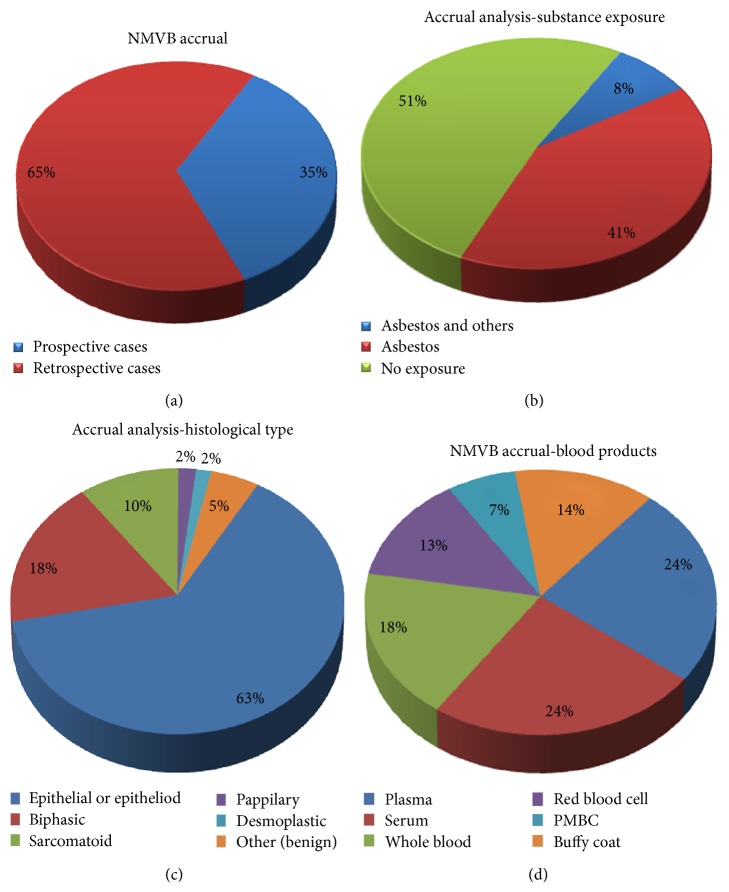
(a) presents the distribution of retrospective and prospective mesothelioma cases of NMVB biorepository. (b) presents the breakup of NMVB accruals as per harmful substance exposure. (c) presents the breakup of NMVB accruals as per histological type of mesothelioma specimen collection. (d) presents the types of blood products available to research community. NMVB resource processes blood products and stores them as per standardized banking protocols.

The NMVB resource has accrued more than 1100 mesothelioma retrospective and prospective cases. The resource also provides more than 1300 biospecimens including over a thousand tumor blocks that are accrued from surgical resections and biopsies and also includes blood products as well (plasma, serum, whole blood, red blood cell, PMBC and buffy coat). This collection is made possible by the joint effort of all the collaborative sites. At the end of 2011 we are hoping to have over 1100 annotated cases of pleural, peritoneal, and pericardial mesothelioma specimens including fresh frozen, paraffin embedded blood products available to the mesothelioma research community (Figure 2). In addition, the resource provides three distinct Tissue Microarrays (TMAs) that have been made available to investigators along with de-identified clinicopathology and follow-up data in excel and XML format [1, 17]. 

### 3.1. NMVB Database

**Figure 3:**
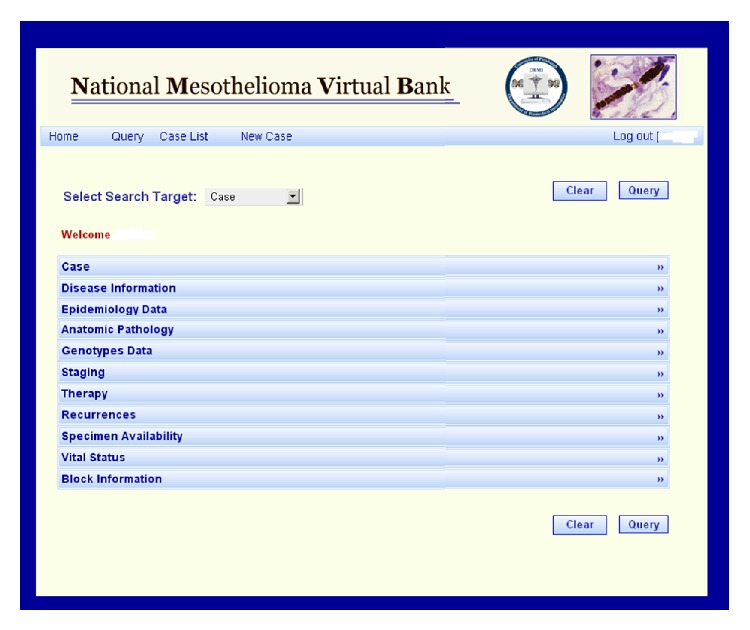
NMVB password protected database. It is only accessible to the data manager for the annotation process and an approved investigator through REP process. Approved investigator can see only de-identified case level clinical information.

The NMVB database consists of two separate interfaces. First one is password protected data entry and query interface (Figure 3). This interface allows data managers at each of the collaborative sites to enroll mesothelioma cases and enter de-identified information at patient's demographic epidemiology, pathology, and follow-up data sets in control access environment. This prohibits data managers of one site to enter and edit the case information of other collection sites. The data managers can create their own case list as per their account assignment and export results as per selected data elements. The NMVB database also provides access to the query tool that is secured and password protected and only investigators approved by IRB and Scientific Review Committee are capable to access the database. It permits end-users to develop their own case lists for their applications and query the data related to the specimen cases they have received from the resource for their approved studies. The query results show only de-identified datasets associated with each approved case through disease and specimen pre-defined views of the data [5].

**Figure 4:**
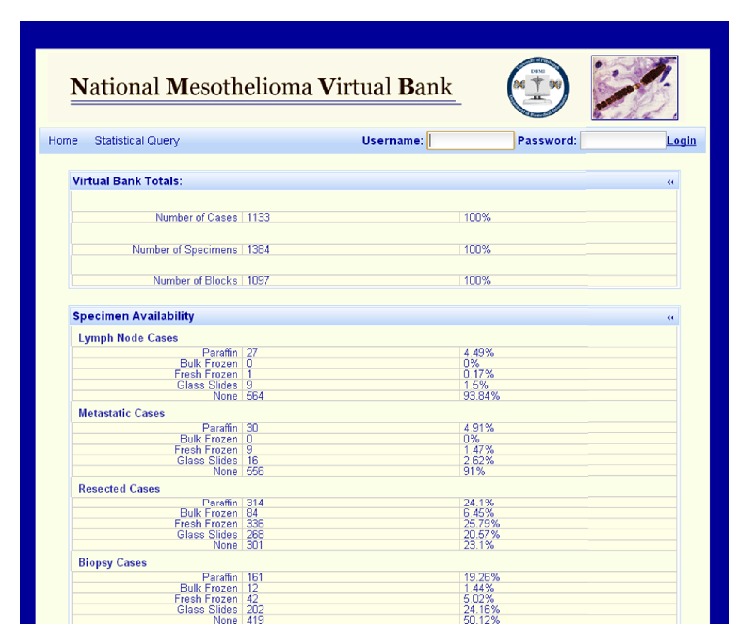
NMVB statistical database provides composite results to users upon querying the NMVB database.

The NMVB statistical database/public view interface is available to the research community (Figure 4) (be consistent with your style). The statistical database for public query provides summary statistics on all the mesothelioma cases and their associated biospecimens stored into NMVB resource. The result page shows the number of cases, specimens, and blocks in the database that match with query criteria of the investigator and general statistics on a limited number of data elements. By utilizing this public view database, the investigator could be able to gain enough information to decide if the resource has sufficient biospecimens to fulfill his or her research needs [5].

### 3.2. NMVB Tissue Microarray (TMA)

In the era of genomics and proteomics, NMVB has realized the distinct utility and growing interest of mesothelioma research community and developed three distinct tissue microarrays. Each of the collaborating site constructed TMA from the cases that are collected and stored at their sites. TMA is a type of tissue assay that permits the investigator to visualize and study tissues from hundreds of patients. The detailed information is available via NMVB website and investigator can obtain data associated with each TMA in excel and XML format. In addition, we have provided whole slide image of each TMA slide so investigator can visualize each core at 20*x* to 40*x* magnifications. A brief description on each tissue microarray is as shown in (Table 1) [6].

### 3.3. Process of Utilization of Resource

The main objective of the NMVB resource is to provide high quality and well-annotated mesothelioma specimens to the research community. In the last seven years, NMVB has provided hundreds of mesothelioma specimens (tissue and blood) and dozens of Tissue Microarray slides. The investigator can obtain the specimen and data by submitting a letter of intent (LOI) electronically via NMVB website or send the LOI via e-mail to the project coordinator. The detailed information about the availability of specimen and obtaining the specimen from resource is available on the NMVB website (http://www.mesotissue.org/). All submitted LOIs are initially evaluated at the coordinating site that includes the authentication of investigator by verifying investigator's credentials and IRB approval of the study from the applicant's institution. After the initial review, LOI is forwarded to REP which determines the validity and justification of the use of specimens from the resource. The REP provides its decision and the applicant is notified about the decision on the LOI. If REP approves the request, LOI is processed further and material transfer agreement is also processed prior to shipping the requested specimen. If the request is denied, a formal letter with feedback from REP is sent to the applicant and a provision is granted to the applicant to resubmit the LOI after addressing the concerns that are pointed out by the REP [18].

### 3.4. Marketing of Resource

The resource has adopted various types of media resources to advertise the NMVB biospecimens and services to the research community. The NMVB website provides general information about the resource and different types of specimens available to the research community. We circulate a quarterly announcement letter via mass marketing e-mailing to investigators that invites them to utilize the resource. Advertisement is made through specialty scientific journals, research society newsletters, fliers at research meetings, posters and podium presentations, marketing booths at scientific research meetings, and free listings in journals and websites.

## 4. Discussion

All collaborative sites of NMVB resource collect and harbor specimens locally within their own tissue banks, while the data and information related to these specimens maintain a centrally located bioinformatics and data management system that is accessible to the members of the network and research community. The resource has established and implemented the decentralized collection and storage with a centralized data management model successfully. During the last six years, the resource has faced many challenges and barriers and yet has overcome them with collaborative efforts and experiences from the previous tissue banking infrastructure development and implementations.

One of the key issues that arose after the initial development of the NMVB resource was to consent mesothelioma patients prospectively. To consent patients prospectively, there was a need of creating a clinical research team that has the ability to approach the patients preoperatively and postoperatively to consent and enroll them in the NMVB study. The clinical research team consists of a research nurse coordinator, thoracic or gastroenterology oncology surgeons, and physicians. A well-defined and highly effective workflow (described above) for prospective enrollment and specimen collection has been established and implemented for all of the NMVB collaborative sites. Each site has developed its own consent form to consent and enroll mesothelioma patients for NMVB prospectively and obtained approval from their local IRB. Efforts have been successful in the placement of a health assessment questionnaire to obtain patient's demographic, previous medical conditions, occupation, exposure to harmful substances including asbestoses, and pertinent family history. This questionnaire is handed over to the patients at the time of consent and requests from the patient to complete it at their convenience. The research nurse coordinator is responsible for the collection of questionnaire and sending it to data management team so they can store the patient's questionnaire information in the NMVB database. During the process of prospective enrollment, we have realized that even with enormous team efforts and a well-organized workflow of prospective collection, we have not been able to successfully consent a considerable fraction of the mesothelioma patients. There are several reasons to explain the less than optimal completion of the consent process such as the shortage of trained research staff at the consenting sites, patient refusal for consent, and a lack of follow-up to consent. To address these issues and improve the enrollment, NMVB principle investigators and working group members have now established a workflow to identify missing mesothelioma cases at each site and enroll them into NMVB resource. The data manager at each collaborative site searches for mesothelioma cases which are seen at their corresponding sites in the previous years. The data managers cross check these cases with their prior mesothelioma enrollment in NMVB database. The data managers then prepare a list of nonconsented cases at their site and identify the vital status on them. The patients with vital status alive are contacted by their treating physician and surgeons to consent for the NMVB study. The deceased patients are also enrolled in NMVB study as per IRB exempt approval. 

Data managers at each collaborative site carry out the clinical annotation process and data extraction process. These processes are performed manually from data sources that include medical record systems, pathology reports, cancer registry, and laboratory information systems. The collected data is then standardized, de-identified, and stored in the database as per the approved study protocols. There are many challenges in data collection, processing, and storage that we have faced and resolved in the last six years. In data collection process, the most common issue we dealt with was to identify the same patient in different hospital data sources. This issue is resolved by developing a patient tracking system that links patient's common identifiers with study identification number through keeping honest broker log sheet. By employing this solution, we are able to identify the same patient across various hospital sources, collecting the accurate data in precise context and reducing the chances of errors in data recording significantly. This solution addresses the challenges in manual data collection and entry into database by the data managers. 

To protect patient health information, a de-identification process is adopted which includes removing patient's clinical identified data sets from the medical records and separating these from the research de-identified data sets. This process is done by honest brokers who act as the information barriers between clinically identified data sets and the de-identified research data sets. The honest broker collects clinical data, identifies the patient identifiers, separates them by assigning the research de-identified numbers, and finally making the de-identified data set available to the community. 

NMVB has adopted a very comprehensive and robust specimen processing and storing protocol and has enforced it at all collaborative sites. The tissue bank technicians are notified for the procurement of tissues/biospecimens. They are responsible for responding to pages from the operation room and doctor's office. The technician must respond quickly and professionally. The technician takes the tissue into the frozen section room or the pathology gross dissection room and records warm ischemic time, weigh the tissue, and properly record it on the specimen sheet. The technician notifies the pathologist on call that tissue is available for banking and ready for gross evaluation. The technician assigns the NVMB study number to the case and records subjects name and NMVB number in the NMVB study log. The samples are divided into three pieces and processed. The first piece of tissue sample is placed in a Bitran bag and freezed in isopentane (bulk frozen). The second one is placed in formalin to be processed to freshed frozen paraffin embeded (FFPE). The third one is placed OCT (embed) and freezed in isopentane. This process is repeated for each tissue sample and label the sample A1, A2, and A3, respectfully, for as many pieces that you receive. The second sample on the same subject would read B1, B2, B3, and so on. The bulk frozen and the OCT tissue samples are placed in a labeled plastic bag in the –80°C freezer in the freezer repository. 

NMVB is a robust informatics supported mesothelioma biorepository that not only provides annotated biospecimen to the research community but also provides a unique platform for enabling collaborative research. To accomplish this goal, NMVB will also participate as a pathology informatics core in a recently submitted Specialized Program of Research Excellence (SPORE) mesothelioma grant supported by National Cancer institute (NCI) [19]. NMVB will also assist in establishing a standardized tumor bank infrastructure and regulatory compliance frameworks for collaborating institutions that will participate in SPORE Mesothelioma project as a specimen and data sites. These new collection sites will collect annoated mesothelioma biospecimen for the SPORE Mesothelioma project and link them to the established NMVB collection sites (as described above in this paper). This partnership will enable mesothelioma research community to have access to a larger resource of well-annotated mesothelioma specimens through the infrastructure of the NMVB distribution network. This expansion will allow for a large and diverse set of patient specimens to be shared for translational and applied research for the mesothelioma community.

## 5. Conclusions

The NMVB acts as a central resource for mesothelioma annotated biospecimens for investigators using experimental methods in translational, histopathology, validation, and outcomes measures at national and international levels. This NMVB resource has been developed on a robust and efficient informatics infrastructure that facilitates a well-crafted workflow management, a standardized collection processes and a subsequent detailed clinical and pathological annotation of diverse cases across multiple collaborative sites. This resource provides a standard manual of operations and procedures, a histopathology guide, and a database with common data elements for characterization of mesothelioma biospecimens, multimodal datasets, and a quality assurance and control process that is intimately integrated within this process.

The NMVB web-based query tool allows researchers to search clinically annotated biospecimens efficiently that are pertinent to their research areas. The web-based query tool ensures the patient's health information protection by disclosing only de-identified data with Institutional Review Board (IRB) and scientific review committee approved standards and protocols. In addition, NMVB website provides an online process of requesting access to using mesothelioma biospecimen, a statistical query tool for the public view and access to approved investigator query tool to potential investigators. The biospecimens disbursement and database access to patient de-identified clinical data is granted after Research Evaluation Panel (REP) approval. In fact NMVB provides a foundation for the development and implementation of an integrated tissue banking program and facilitates other tissue banking efforts through the members of the collaborative resource and their associated publications.

## Figures and Tables

**Table 1:** NMVB resource provides three unique and customized tissue microarrays to the research community. This table provides a brief description about the TMAs resource. NYU (New York University), U. Penn (University of Pennsylvania), and UPMC (University of Pittsburgh Medical Center).

	TMA 1 (UPMC)	TMA 2 (U. Penn)	TMA 3 (NYU)
Number of cases	40	54	37
Benign	4	0	1
Malignant	36	54	35
Metastatic	3	0	1
Primary/metastatic	1	3	10
Control	0	31	5
Number of cores	135	291	132
Number of slides	1	4	3
